# *CpGcluster*: a distance-based algorithm for CpG-island detection

**DOI:** 10.1186/1471-2105-7-446

**Published:** 2006-10-12

**Authors:** Michael Hackenberg, Christopher Previti, Pedro Luis Luque-Escamilla, Pedro Carpena, José Martínez-Aroza, José L Oliver

**Affiliations:** 1 Dpto. de Genética, Facultad de Ciencias, Universidad de Granada, Spain; 2 Dpto. de Ingeniería Mecánica y Minera, Universidad de Jaén, Spain; 3 Dpto de Física Aplicada II, Universidad de Málaga, Spain; 4 Dpto. de Matemática Aplicada, Facultad de Ciencias, Universidad de Granada, Spain; 5 Dept. of Molecular Biophysics, German Cancer Research Center, Heidelberg, Germany

## Abstract

**Background:**

Despite their involvement in the regulation of gene expression and their importance as genomic markers for promoter prediction, no objective standard exists for defining CpG islands (CGIs), since all current approaches rely on a large parameter space formed by the thresholds of length, CpG fraction and G+C content.

**Results:**

Given the higher frequency of CpG dinucleotides at CGIs, as compared to bulk DNA, the distance distributions between neighboring CpGs should differ for bulk and island CpGs. A new algorithm (*CpGcluster*) is presented, based on the physical distance between neighboring CpGs on the chromosome and able to predict directly clusters of CpGs, while not depending on the subjective criteria mentioned above. By assigning a *p-value *to each of these clusters, the most statistically significant ones can be predicted as CGIs. *CpGcluster *was benchmarked against five other CGI finders by using a test sequence set assembled from an experimental CGI library. *CpGcluster *reached the highest overall accuracy values, while showing the lowest rate of false-positive predictions. Since a minimum-length threshold is not required, *CpGcluster *can find short but fully functional CGIs usually missed by other algorithms. The CGIs predicted by *CpGcluster *present the lowest degree of overlap with Alu retrotransposons and, simultaneously, the highest overlap with vertebrate Phylogenetic Conserved Elements (PhastCons). *CpGcluster's *CGIs overlapping with the Transcription Start Site (TSS) show the highest statistical significance, as compared to the islands in other genome locations, thus qualifying *CpGcluster *as a valuable tool in discriminating functional CGIs from the remaining islands in the bulk genome.

**Conclusion:**

*CpGcluster *uses only integer arithmetic, thus being a fast and computationally efficient algorithm able to predict statistically significant clusters of CpG dinucleotides. Another outstanding feature is that all predicted CGIs start and end with a CpG dinucleotide, which should be appropriate for a genomic feature whose functionality is based precisely on CpG dinucleotides. The only search parameter in *CpGcluster *is the distance between two consecutive CpGs, in contrast to previous algorithms. Therefore, none of the main statistical properties of CpG islands (neither G+C content, CpG fraction nor length threshold) are needed as search parameters, which may lead to the high specificity and low overlap with spurious Alu elements observed for *CpGcluster *predictions.

## Background

Given the inherent mutability of methylated cytosine, the human genome has only a fraction (≈ 20%) of the CpG dinucleotides expected on the basis of its G+C content [1,2]. However, the resulting scarcity of CpGs is not uniform throughout the chromosome: there are many DNA tracts (CpG islands or CGIs), totaling 1% of the genome, where CpGs are abundant [3-6]. The lack of methylation at CGIs, together with their elevated G+C content relative to the genome average, results in a frequency of CpG dinucleotides that is about 10-fold higher than in bulk DNA [5,6]. About 60% of all genes have a CGI, normally unmethylated, in their promoter region [2,6,7]. However, in some physiological or pathological situations promoter-associated CGIs can be methylated, then provoking a change in the expression of the associated gene [8-11]. The maintenance of a particular genomic pattern of methylated CpGs provides an epigenetic means for differential regulation of gene expression [2,7,12].

Approximately 80% of all CpGs are methylated in human and mouse genomes, which makes the hypomethylated and GC-rich CGIs an outstanding genomic property. Given their putative function in gene regulation and their importance as genomic markers in promoter prediction, over recent years there has been a considerable effort to predict CGIs *in silico*. Current algorithms (*newcpgreport *[13]; *cpg *[14]; *CpGProD *[15]; *CpGIS *[16,17]; *CpGIE *[18]; *CpGED *[19]) rely on the *ad hoc *thresholds of length, CpG O/E ratio and G+C content early defined by Gardiner-Garden and Frommer [20]. These three thresholds lead to a parameter space which is relatively large and difficult to explore completely. Consequently, in many publications, these parameters have been fine-tuned in different ways -for example, to filter out spurious Alu elements or restricting the prediction to putative promoter CGIs. However, in every fine-tuning, "valid" CGIs also become filtered out, as a consequence of using the same parameters for both prediction and filtering; this suggests the use of different parameters in both steps, as proposed below in the *CpGcluster *algorithm. Another shortcoming, shared by the algorithms using the conventional moving-window approach (*newcpgreport*, *CpGProdD*, *CpGIS *and *CpGIE*), but not by the *cpg *script (which uses compositional segmentation) or *CpGED *(which uses a sliding double window), is that the island boundaries cannot be accurately defined to single base-pair resolution. As is well known (see, for example [21]), the methods using a moving window add another level of subjectivity in choosing both the length of the window and the step size. Taking this problem into account, the algorithm *CpGcluster *is able to predict the island boundaries to a single base-pair resolution by definition.

Bulk CpGs are thought to be in a dynamic equilibrium between the decay of methylated CpGs and the generation of new ones due to point mutations [2]. This is a random process and therefore the CpG distance distributions should be strikingly different for bulk and island CpGs, which motivated our approach. In particular, the distances between consecutive bulk CpGs, as the result of a random process, should follow the geometric distribution, while the distance distribution for in-island CpGs must contain information on the high local clustering. Taking advantage of this high local clustering of CpG dinucleotides at CGIs, *CpGcluster *directly predicts clusters of CpGs on the chromosome. Predicted clusters with high enough statistical significance can then be identified as CGIs (see Methods).

## Results

### Benchmarking *CpGcluster*

The accuracy of *CpGcluster *was evaluated by comparing it to five commonly used CGI finder programs (Table 1). To benchmark the programs, a set of test sequences containing experimentally determined CGIs in a random background was assembled, as described in the Methods section. As in the gene-finding field [22], the accuracy of a prediction was measured by comparing the predicted island value (island or non-island) with the true island value for each nucleotide along the test sequence, then deriving estimates for Sensitivity (Sn, the proportion of island nucleotides that have been correctly predicted as islands), Specificity (Sp, the proportion of predicted island nucleotides that are actually islands; this measure is also known as Positive Predictive Value, or PPV) and the Correlation Coefficient (CC, a single scalar value that summarizes Sn and Sp as a measure of global accuracy). The averaged values over ten test sequences, each with 400 experimental islands randomly distributed over a randomized sequence, are shown in Table 1. When the threshold distance was set to the median of the observed distribution, *CpGcluster *showed moderate values for Sn, while reaching the highest ones for both CC and Sp. The high specificity achieved by our algorithm indicates that it has the lowest rate of false-positive predictions (i.e. only 2.4% of the predicted nucleotides turned out actually not to be part of a CGI).

**Table 1:** Benchmarking of *CpGcluster*

Program	Sn ± SD	Sp ± SD	CC ± SD	Hit* [%] ± SD
*Newcpgreport*	0.545 ± 0.002	0.973 ± 0.002	0.725 ± 0.005	87.000 ± 0.540
*CpGProD*	0.918 ± 0.003	0.657 ± 0.003	0.772 ± 0.006	94.675 ± 0.808
*CpGIS*	0.832 ± 0.003	0.756 ± 0.007	0.789 ± 0.013	86.675 ± 1.528
*CpGIE*	0.910 ± 0.002	0.667 ± 0.003	0.775 ± 0.006	94.650 ± 0.810
*CpGED*	0.819 ± 0.013	0.584 ± 0.004	0.685 ± 0.005	84.075 ± 1.191
*CpGcluster *(d_*t *_= median, or 44 bp)	0.655 ± 0.003	0.976 ± 0.005	0.797 ± 0.009	95.475 ± 0.870
*CpGcluster *(d_*t *_= 75^th ^percentile, or 94 bp)	0.866 ± 0.006	0.832 ± 0.009	0.846 ± 0.006	95.050 ± 0.643

*Hit: Percentage of true islands overlapping (by at least one nucleotide) with predicted islands.

Average accuracy values (Sn, Sp and CC) of CpGcluster and other five CGI finders over 10 test sequences, each one with 400 experimental CGIs. Default parameters values, as recommended in the corresponding publications, were used for each program.

For these results, the median distance between neighbor CpGs and a *p-value *cutoff of 10^-5 ^were used to run *CpGcluster*. As shown in the last row of Table 1, the raising of the distance threshold to the 75^th ^percentile, thereby obtaining longer islands, increased sensitivity by more than 20% while only minimally improving overall accuracy. However, this led to a smaller fraction of CGI overlapping with PhastCons (shown below). On the other hand, lowering the *p-value *threshold beyond 10^-5 ^slightly increased Sp but also clearly decreased Sn, thus lowering overall global accuracy (not shown). Consequently, the median distance was used as the only parameter for the island prediction and the 10^-5 ^cutoff for the filtering in all subsequent analyses.

Finally, we examined another accuracy indicator, the Hit percentage, which gives the proportion of experimental CGIs which have been at least partially overlapped by the predicted islands. Table 1 shows that *CpGcluster *"hits" a higher number of islands than any other algorithm. This highest partial overlap (at least the core region of the CGI is predicted), together with the highest specificity mentioned above, might indicate an advantage of *CpGcluster *over the other tested algorithms.

### Statistical analysis of predicted islands in human and mouse genomes

Basic statistics of the CGIs predicted by *CpGcluster *in the human (hg17) and mouse (mm7) genome assemblies are shown in Table 2. For comparison, this Table also includes an analysis of the islands predicted by *CpGProD*. The number of CGIs predicted by *CpGcluster *in both genomes is higher, and their average length shorter than those predicted by *CpGProD *or the ones previously reported in the literature (see, for example, [6]). Some of the short CGIs predicted by *CpGcluster *might be spurious, but some others may play a true functional role (see below). The spurious fraction may be due, for example, to the presence of arginine-rich exons, which are then rich in CGN codons, and therefore are prone to erroneous identification as CGIs. Comparing our prediction to annotated exon boundaries, we estimated this fraction to be relatively low (only about 3.4% of the predicted CGIs in the human genome correspond actually with exons).

**Table 2:** Basic statistics of *CpGcluster *and *CpGProD *islands

	**hg17**		**mm7**	
		
	***CpGcluster***	***CpGproD***	***CpGcluster***	***CpGproD***
Genome length (without N-runs, bp)	2.85E + 09	2.85E + 09	2.51E + 09	2.51E + 09
Total number of CpGs	28,073,991	28,073,991	20,967,593	20,967,593
CpG-dinucleotides in CpG-islands (%)	4,489,575 (15.99)	4,323,799 (15.40)	2,708,986 (12.92)	2,215,608 (10.57)
Number of islands predicted	197,727	76,793	117,373	40,171
*Island coverage (%)	1.90	2.81	1.47	1.65
Island length (bp):				
Average	273.5 ± 246.7	1043.8 ± 761.7	314.0 ± 293.8	1030.3 ± 560.0
Minimum	8	500	8	500
Maximum	7,774	42,276	5,618	9,288
Average island GC-content (%)	63.76 ± 7.51	54.58 ± 6.12	61.58 ± 10.03	54.62 ± 5.17
Average CpG O/E ratio	0.855 ± 0.265	0.636 ± 0.089	0.956 ± 0.428	0.652 ± 0.103
Average CpG-density	0.087 ± 0.041	0.047 ± 0.016	0.097 ± 0.084	0.048 ± 0.015

*Percentage of the genome covered by the CpG-islands.

Basic statistics of the CpG-islands predicted by *CpGcluster *and *CpGProD *in the human (hg17) and mouse genomes (mm7).

The hypothesis that some short CGIs could be truly functional is based on the fact that many known functional CGIs are shorter than commonly assumed -the extreme example being *Xenopus *CGIs, which are known to be shorter and have a lower G+C content than the CGIs found in mammals [23]. However, short and functional CGIs exist also in the human genome. One example concerns the CGI of the human tissue-specific SERPINB5 gene. The promoter of this gene is associated with a GC-rich region that, while fulfilling the conventional %G+C and CpG fraction defining criteria for CGIs, is significantly shorter than the average [24] and consequently goes unnoticed in most annotations [2]. To our knowledge, *CpGcluster *is the only algorithm capable of catching the core of this fully functional CGI [see Additional file 1]. A second example refers to MAGE genes, which are found as antigens in a wide variety of tumors, and become methylated during normal mammalian development. They have a CpG-rich region 300–650 bp long at their 5' end that, although shorter than average CGIs, remains nonmethylated in sperm but methylated in somatic tissues, where the genes are not expressed. Therefore, these genes represent clear examples of tissue-specific genes that use DNA methylation as a primary mechanism for their regulation [25]. The ability to detect the CpG-rich regions enabling this type of regulation is an important measure of quality for any CGI finder and was tested on ten MAGE genes having known TSS (Table 3). Our algorithm detected CGIs in eight of the ten MAGE genes analyzed, while the number of islands reported by the other programs in this gene set was significantly lower.

**Table 3:** Overlap with PhastCons and MAGE genes

	Overlap with TSS of MAGE genes		% of overlap with	
Program	#CGI	Average length ± SD	Alus	PhastCons

*newcpgreport*	2	271.0 ± 18.4	19.49	23.73
*CpGProD*	3	1,314.3 ± 525.1	23.40	13.31
*CpGIS*	3	800.0 ± 243.3	10.52	20.59
*CpGIE*	3	1,093.0 ± 476.1	23.99	14.00
*CpGED*	2	730.5 ± 320.3	15.32	15.82
*CpGcluster*	8	258.3 ± 100.8	6.79	28.53

Predicted CpG islands in 10 MAGE genes (left part) and percentage of overlap between the CpG-islands predicted in the human chromosome 22 and both Alu retrotransposons and PhastCons (evolutionarily conserved elements).

The minimum length of a functional CGI is a difficult question, but insights can be derived from recent advances in mapping functional promoters. The shortest island in our prediction which overlaps with a TSS from DBTSS is 33 bp in length. When functional promoters are determined through ChIP-on-chip technology [26], that length goes down to 13 bp. Finally, when promoters are determined by using the cap analysis of gene expression (CAGE) approach [27] the minimum island length is just 11 bp long, thus approaching the minimum lengths observed in both DerLab and CpGcluster databases. Thus, it seems that even very short islands may be functional. Also, it bears mentioning that short islands (<200 bp) predicted by *CpGcluster *which overlap with a TSS also show a very high degree of overlap (37%) with conserved elements (see below), thus suggesting probable biological relevance.

Further insight into the possible role of short CGIs is suggested by the recent finding of CpG "islets", genomic regions that are not conventionally classified as CpG islands because of their short length (<200 bp), but have a GC content and observed-to-expected CpG ratio that is characteristic of a CpG island. CpG islets may be non-methylated, corresponding to sites of active transcription and/or boundaries that separate major centromeric chromatin sub-domains [28].

All in all, these data support the possibility that genomic tracts with GC content and CpG Obs/Exp ratios typical of CGIs, but below the detection threshold of conventional CGI finders, may have a functional role in the genome. CpGcluster represents a new tool that may help to uncover such regions.

### Minimal overlap between CGIs predicted by *CpGcluster *and Alu retrotransposons

A major source of uncertainty in CGI prediction is the interference of Alu retrotransposons. These elements, abundant in primate genomes, have often been falsely identified as CGIs by conventional CGI finders. To cope with this problem, some authors [15,16,18] have proposed a simple increment in the value of some of the thresholds used. The drawback of such a strategy is that some CGIs associated with genes would also be excluded under these more stringent criteria. Even so, the fraction of Alu overlap shown by the islands predicted by most programs is still rather large, while *CpGcluster's *CGIs demonstrate the least amount of overlap with Alu elements (Table 3). We wish to stress especially that *CpGcluster *does not need any minimum-length criterion to exclude a higher proportion of Alu elements than any of the previously existing algorithms tested.

### Highest degree of overlap between *CpGcluster *islands and phylogenetic conserved elements (PhastCons) from vertebrates

Functional genomic elements, being under natural selection, are expected to be highly conserved during evolution. Therefore, if the predicted CGIs truly play a functional role, they should show a high degree of overlap with vertebrate PhastCons [29]. Taking advantage of the 'most conserved' track (based on the best-in-genome pairwise alignments between human and other seven vertebrate genomes) at UCSC Genome Browser [30], we computed the percentage of overlap between PhastCons and the CGIs predicted by the different finders. As seen in Table 3, the islands predicted by *CpGcluster *show the highest degree of overlap with PhastCons, thus indicating that our algorithm predicts a higher proportion of evolutionarily conserved, functionally relevant CGIs than does any other tested algorithm.

### Promoter and CpG island co-location

For a further quality assessment for the islands predicted by *CpGcluster*, we assigned them to five classes according to their co-location with annotated genes from the RefSeq database [31]. The classification proposed by Ioshikhes and Zhang [32] was improved by using exon boundaries (instead of absolute positions) to define the different classes. Accordingly, we divided CGIs into five classes defined as follows: L1, the island overlaps with the TSS; L2, the island does not overlap with the TSS but is located somewhere between 2 kb upstream of the TSS and the end of the first exon; L3, the island is located somewhere between the end of the first exon and the start of the last exon; L4, the island is located between the start of the last exon and 2 kb downstream of the Transcription End Site (TES); NG, the island is outside the gene environment.

Most of the islands predicted by *CpGcluster *are located outside of the gene environment (Table 4). Only 56750 (or 28.7%) in humans and 40348 (or 34.9%) in mice are within or around the genes. Note, however, that the curate samples of RefSeq genes used in elaborating this table represent less than half of the existing genes in both species. When we analyzed the entire RefSeq database without any filtering, these percentages rose to 53.4% and 47.2%, respectively. When Genscan gene predictions, another well-known gene-finder track available at the UCSC Genome Browser, were considered, the percentages rose to 83.4% and 82.2%, respectively. These results indicate that a substantial fraction of the CGIs predicted by CpGcluster may overlap the putative, non yet confirmed genes predicted by this popular gene-finder.

**Table 4:** Location of CpGcluster islands

						P-values			
							
Class*	# CpG islands	Length ± SD	CpG Density ± SD	Obs/Exp Ratio ± SD	%GG ± SD	P25	Median	P75	PhastCons overlap (%)
**Human:**									
L1	6,775	672.3 ± 398.7	0.10 ± 0.02	0.89 ± 0.13	68.4 ± 5.7	1.43E-66	1.53E-40	8.43E-23	29.73
L2	16,709	256.3 ± 213.1	0.09 ± 0.04	0.89 ± 0.23	64.6 ± 7.8	2.61E-14	5.42E-09	7.27E-07	21.56
L3	29,386	230.5 ± 173.0	0.08 ± 0.04	0.84 ± 0.27	63.1 ± 7.1	1.17E-10	9.94E-08	1.86E-06	14.97
L4	3,880	247.8 ± 212.8	0.09 ± 0.04	0.85 ± 0.23	65.8 ± 7.3	2.12E-12	3.43E-08	1.31E-06	28.04
NG	140,977	266.0 ± 238.2	0.09 ± 0.04	0.85 ± 0.27	63.5 ± 7.5	1.44E-12	2.43E-08	1.25E-06	14.06
**Mouse:**									
L1	8,090	745.7 ± 373.8	0.09 ± 0.02	0.83 ± 0.13	65.3 ± 5.2	2.50E-64	1.20E-40	7.20E-24	35.69
L2	10,219	302.7 ± 257.4	0.09 ± 0.07	0.92 ± 0.38	62.0 ± 9.3	8.60E-15	9.15E-09	8.69E-07	38.80
L3	18,734	230.6 ± 190.7	0.10 ± 0.09	0.97 ± 0.45	61.4 ± 10.4	9.91E-10	2.04E-07	2.29E-06	34.99
L4	3,305	284.6 ± 232.6	0.08 ± 0.06	0.87 ± 0.35	61.3 ± 8.3	1.61E-11	7.17E-08	1.58E-06	49.87
NG	75,419	284.0 ± 257.6	0.10 ± 0.09	0.98 ± 0.45	61.1 ± 10.5	2.54E-12	2.92E-08	1.29E-06	20.45

*See text for a description of the different classes.

Co-location of CpG islands predicted by *CpGcluster *and genes in human (hg17) and mouse (mm5) genomes.

Table 4 also shows that both in humans and in mice *CpGcluster *predicts drastically different islands as a function of genomic location: promoter CGIs (L1) are longer and have lower *p-values *than do the rest of the classes. Another important observation is that both in humans and mice, promoter CGIs are much richer in vertebrate PhastCons elements than are non-genic islands (NG).

In addition, two surprising observations were made in the mouse genome: 1) promoter islands have smaller CpG densities and CpG fractions than non-genic islands have; and 2) L4 islands, located mainly in the 3' untranslated regions (3' UTRs), show a high proportion of PhastCons overlap. It is well known that when mouse and human orthologous genes are compared, the mouse line shows a net loss of CpG islands [6]. This probably indicates a higher "pressure" on CGIs in mice, which may account for these findings.

For a comparison, we also analyzed the co-location of genes with the CGIs predicted by *CpGProD *(Table 5). As shown in Table 2, the statistical properties of the CGIs predicted by this finder are quite similar for mouse and human. However, as shown in Tables 2 and 4, *CpGcluster *predicts islands with striking differences between human and mouse, especially when looking at the co-location with genes. For example, mouse CGIs overlapping with TSS have lower CpG densities and Obs/Exp ratios than non-genic islands. We interpret these as being the true statistical properties of those islands (overlapping with a TSS), as *CpGcluster *does not predetermine these values in the detection process.

**Table 5:** Location of CpGProD islands

						P-values			
							
Class*	# CpG islands	Length ± SD	CpG Density ± SD	Obs/Exp Ratio ± SD	%GG ± SD	P25	Median	P75	PhastCons overlap (%)
**Human:**									
L1	7,310	1,831.5 ± 875.8	0.06 ± 0.01	0.74 ± 0.09	59.3 ± 5.0	2.30E-78	4.20E-51	7.06E-33	21.01
L2	3,542	933.0 ± 599.3	0.04 ± 0.01	0.62 ± 0.08	53.6 ± 5.8	1.40E-17	1.35E-09	3.91E-07	13.66
L3	10,582	814.5 ± 436.2	0.04 ± 0.01	0.61 ± 0.07	53.4 ± 5.6	1.10E-13	3.68E-09	4.36E-07	10.66
L4	1,218	1,097.2 ± 775.8	0.05 ± 0.02	0.63 ± 0.08	56.2 ± 6.8	6.79E-32	5.47E-12	1.35E-07	16.63
NG	54,141	988.3 ± 739.8	0.05 ± 0.02	0.63 ± 0.09	54.2 ± 6.1	2.30E-21	2.03E-10	1.33E-07	12.15
**Mouse:**									
L1	7,938	1,463.4 ± 576.0	0.06 ± 0.01	0.72 ± 0.11	58.1 ± 4.2	8.30E-67	3.86E-43	9.89E-28	27.82
L2	1,764	1,007.7 ± 560.1	0.05 ± 0.01	0.64 ± 0.11	55.0 ± 5.2	3.88E-31	2.65E-16	3.32E-10	34.15
L3	4,050	807.2 ± 374.0	0.04 ± 0.01	0.61 ± 0.08	53.1 ± 4.5	7.05E-17	3.52E-11	1.19E-08	26.29
L4	716	997.4 ± 501.5	0.05 ± 0.01	0.64 ± 0.09	55.1 ± 5.1	9.21E-32	1.69E-16	4.21E-10	34.40
NG	24,644	924.4 ± 502.7	0.05 ± 0.01	0.64 ± 0.10	53.2 ± 5.0	4.28E-23	1.84E-13	1.88E-09	17.43

*See text for a description of the different classes.

Co-location of the CpG islands predicted by the program *CpGProD *and genes in human (hg17) and mouse (mm5) genomes.

**Table 6:** Statistics of CpG distances and %G+C in human and mouse chromosomes

	**hg17**			**mm7**		
		
Chromosome	median	mean	%G+C	median	mean	%G+C
1	40	97.7	41.73	63	129.1	41.12
2	46	109.4	40.23	56	116.4	42.07
3	52	119.1	39.69	63	129.9	40.44
4	53	127.1	38.21	52	113.1	42.29
5	49	116.9	39.52	51	108.4	42.51
6	47	112.6	39.60	61	124.8	41.39
7	40	98.6	40.72	53	113.9	43.12
8	46	108.3	40.17	52	109.5	42.35
9	39	96.8	41.36	54	111.5	42.70
10	41	96.2	41.58	54	113.9	41.38
11	41	100.6	41.57	47	101.0	43.82
12	41	101.2	40.80	59	121.2	41.65
13	50	118.1	38.52	57	117.5	41.61
14	42	101.7	40.89	62	126.5	41.10
15	40	92.6	42.21	54	114.5	41.95
16	31	70.9	44.79	61	124.7	40.90
17	29	66.4	45.53	49	106.3	42.61
18	47	109.2	39.79	59	120.2	41.43
19	23	51.8	48.36	49	103.2	42.73
20	36	81.9	44.13	–	–	–
21	37	90.9	40.88	–	–	–
22	28	59.5	47.96	–	–	–
X	52	121.0	39.46	81	168.2	39.22
Y	49	119.9	39.85	67	155.1	39.19

Median and mean distances between CpGs, and average %G+C content for both, human and mouse chromosomes.

## Discussion

*"Stretches of DNA with a high G+C content, and a frequency of CpG dinucleotides close to the expected value, appear as CpG clusters within the CpG-depleted bulk DNA, and are now generally known as CpG islands"*. This original description of CpG islands by Gardiner and Frommer in 1987 [20] formulates the basic idea underlying the present work: CpG dinucleotides appear clustered within the CpG-depleted bulk DNA and these clusters should be able to be associated with CpG islands. In the same work [20], the above authors also proposed a criterion for CpG islands based on thresholds which later became the basic principle of practically all existing CpG island finders. They justify these criteria by assuming that *CpG-rich regions over 200 bp in length are unlikely to have occurred by chance alone*, which points out another important property of CpG islands implemented in this work: the statistical significance. Some years before, McClelland and Ivarie [3] had introduced a Chi-square test to assign a statistical significance to CpG islands. Therefore, our approach is probably more related to the original perception of CpG islands as statistically significant CpG clusters within CpG-depleted regions.

**Figure 1:**
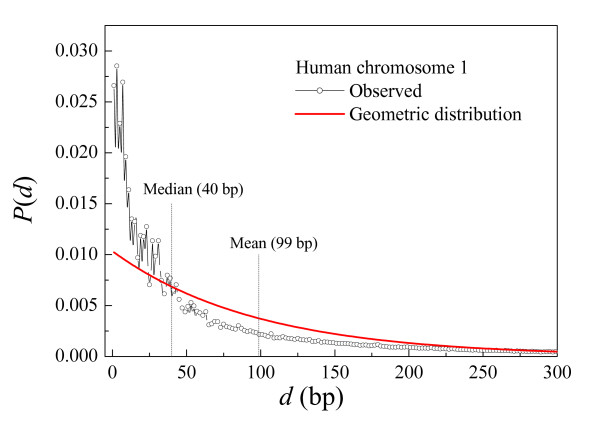
**Probability density function of distances between neighboring CpGs**. Distribution of distances between neighboring CpG dinucleotides in the human chromosome 1. The observed distribution is represented in symbols, while the random expectation corresponding to the geometric distribution [Eq. 1] is represented in a solid line. Note that, in a good approximation, the median separates over-represented distances from under-represented ones.

Both our distance approach (which directly predicts CpG clusters) and the threshold approach are derived from the same original idea stating that the CpGs form clusters in the genome. However, the main disadvantage of any threshold approach is that generally valid CpG islands may become discarded as well, an effect that is aggravated as the dimension of the parameter space grows. In our distance approach, we reduced the parameter space notably, furthermore linking the distance parameter to intrinsic statistical properties of the sequence. The chosen median distance between two CpGs in a given chromosome separates fairly well the CpG clustering from the inter-cluster distances (see Fig. 1) and therefore affords certain objectivity to the choice of this parameter. Note furthermore that the median distance is correlated to the G+C content of the chromosome sequence. The higher the G+C content of the chromosome, the higher the probability that a CpG appears and consequently the lower will be the median distance. In this way, the median distance adjusts itself to the global conditions dictated by the given input sequence. This can hardly be achieved using the conventional large-dimension threshold parameter space and therefore, in previous work, the same threshold values were used indiscriminately for all the chromosomes.

The first consequence of the difference between the distance and threshold approaches is that, on average, *CpGcluster *islands are shorter. However, they show higher mean G+C content, CpG density, and CpG fractions than do any of the other five tested algorithms (Table 2). The lower values shown by these threshold-based algorithms may be an inherited consequence of the general approach shared by most of them. To some extent, the chosen thresholds predetermine the statistical properties of the islands, since these usually become enlarged as long as the thresholds are not violated. This threshold-dependent enlargement in the search process may also lead to the observed over-prediction of CpG islands and high Alu overlap shown by most threshold-based algorithms. On the contrary, *CpGcluster *overcomes this drawback since statistical properties of the CGIs, such as G+C content or CpG fraction are not used as search parameters. Note furthermore that the *p-value *is a crucial filter parameter to sort out spurious Alu elements. Young Alus have *p-values *around 10^-7 ^(with slight variations among chromosomes); therefore, the high substitution rates on the Alu CpG sites produce a fast loss of statistical significance, which explains the low overlap with spurious Alu elements shown by the islands predicted by *CpGcluster*.

Finally, we wish to discuss briefly the lack of any length filter in *CpGcluster *which allows the prediction of extremely short islands and which, at first glance, could be interpreted as a disadvantage. It should be noted that in all of the previous algorithms the length is not used for prediction purposes, and is considered only in the final filtering process. In fact, the original idea of the length threshold was to guarantee that the predicted islands are not just a mere product of chance alone. Instead, we change the length filter by a statistically stricter criterion: the *p-value*. In this way, all predicted CGIs are statistically significant CpG clusters. We are aware that the putative functional CGIs are on average very long (as for example the L1 class in Table 4). However, it is important to stress the conceptual difference between the detection of CpG clusters and the subsequent filtering for a particular subset (e.g. promoter overlapping CGIs). These two steps should be clearly distinguished.

## Conclusion

The distance-based CGI-finder algorithm described here presents three outstanding features: i) all the predicted CGIs start and end with a CpG dinucleotide; ii) all the computations needed use integer arithmetic, thus leading to a fast and computationally efficient CGI finder, and iii) a *p-value *is associated with each of the predicted islands. When compared to other CGI finders,*CpGcluster *is able to predict CGIs with the highest global accuracy and specificity, thus indicating a low rate of false-positive predictions. Short but fully functional CGIs are also predicted by *CpGcluster*. Furthermore, the degree of overlap of predicted CGIs with Alu retrotransposons is minimal, while the overlap with vertebrate PhastCons is maximal. The promoter CGIs predicted by *CpGcluster *also show the highest statistical significance, thus qualifying *CpGcluster *as a valuable tool to distinguish functional CGIs from the remaining islands in the bulk genome.

## Methods

The algorithm CpGcluster presented in this work consists of two main steps: i) a distance-based algorithm searches for clusters of CpGs in the chromosome sequence. ii) a *p-value *is associated with each of these clusters, then predicting as CGIs only those clusters with large enough statistical significance (i.e. for which their *p-values *are below the selected threshold). These two steps are explained in detail in the next subsections.

### CpG cluster-searching algorithm

The cluster-searching method is based on the statistical properties of the physical distances between neighboring CpG dinucleotides on the DNA sequence. In principle, if CpGs are distributed totally at random along the chromosome sequence, the distances between neighboring CpG dinucleotides should follow the geometric distribution:

*P*(*d*) = (1 - *p*)^*d*-1 ^*p *      [1]

where *P*(*d*) represents the probability of finding a distance *d *between neighboring CpGs and *p *corresponds to the probability of CpGs in the sequence, calculated as the ratio between CpGs and the total number of dinucleotides in the DNA sequence.

The working hypothesis behind the cluster-searching algorithm is that the abundant CpGs in CGIs may be separated by shorter distances (thereby forming clusters) than the distances between bulk CpGs, which in principle should follow the geometric distribution [Eq. 1].

**Figure 2:**
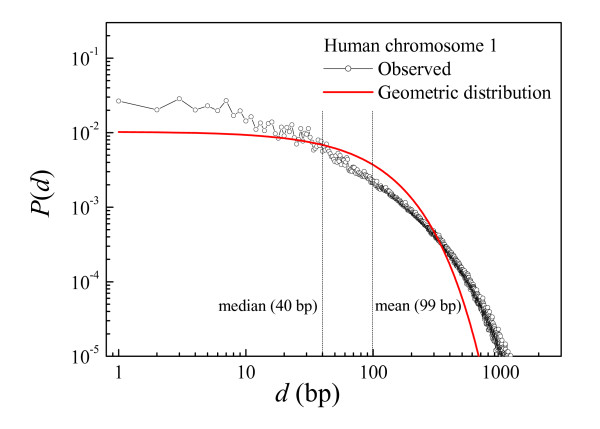
**Probability density function of distances between neighboring CpGs (log-scale)**. The same as in Figure 1, but using logarithmic axis; over-represented large distances can be appreciated.

To test our working hypothesis, in Figure 1 we represent the normalized probability distribution of distances between neighboring CpGs corresponding to human chromosome 1 (Additional files 2, 3, 4, 5 show the normalized probabilities of distances for all the human chromosomes). The median/mean values of CpG distances for all chromosomes are shown in Table 6. Figure 1 shows that short distances are over-represented when compared to the geometric distribution, while intermediate distances are less abundant than the theoretical random expectations. Large distances are also over-represented when compared to the geometric distribution. For a clear display of these features, in Figure 2 we represent the same as in Figure 1, but using logarithmic axis: distances below 40 bp (the median) and above 300 bp are over-represented, while the intermediate values are under-represented. Both facts clearly indicate strong clustering: the abundant short distances separate intra-cluster CpGs while the large distances (also more abundant than randomly expected) separate the clusters themselves.

Therefore, the DNA sequence was scanned looking for the presence of such CpG clusters. The algorithm performs the following steps:

1. The DNA chromosome sequence is scanned for CpG dinucleotides, then recording the positions occupied by the 'C': x_1_, x_2 _... x_N_, N being the total number of CpGs in the sequence. The sequence was usually scanned in the 5' → 3' direction. Trivially, the reverse scan (3' → 5') produces the same results.

2. As a convention, the physical distance separating two neighboring CpGs is defined as:

*d*_*i *_= *x*_*i*+1 _- *x*_*i *_- *1*,       [2]

so that the minimal distance between two neighboring CpGs (i.e. CGCG) is equal to 1.

3. In the course of the scan, the first distance below a given threshold (*d*_*t*_) identifies the first CpG cluster. The threshold *d*_*t *_can be conveniently derived from the distribution of distances between neighboring CpGs in the chromosome sequence. The median distance (Figs. 1, 2) often gives the best results because the median distance of the observed distribution is approximately at the transition point of the over-represented (intra-cluster) small distances and the under-represented intermediate ones. This is not an exclusive property of chromosome 1, as it is shared by all the chromosomes (see Additional files 2, 3, 4, 5), thus indicating that the median distance can be chosen in general as a good threshold (*d*_*t*_).

4. We then try to extend this first cluster downstream (→ 3') by adding the next CpG while the distances are below *d*_*t*_. When a distance exceeding *d*_*t *_is found, the cluster is completed, and the search for a new one continues downstream.

5. Steps 3 and 4 are iterated until all the CpG clusters in the sequence are identified.

Note that this algorithm acquires two important and distinctive features by construction. First, all predicted CGIs start and end with a CpG dinucleotide, which seems appropriate. Secondly, the algorithm uses only integer arithmetic, thus being computationally efficient. No other CGI searching algorithm shares these two important properties.

### Assigning *p-values *to CpG-clusters

Once all the CpG clusters are found in the sequence following the algorithm described above, the next step is to associate a *p-value *with each one – i.e. the probability of such a cluster appearing by chance in a random sequence. Such a probability can be estimated either numerically by a randomization test on the DNA sequence or by means of a theoretical probability function (both cases shown in Additional file 6). For the latter case, the negative binomial distribution (also known as Pascal or Pólya distribution) can be conveniently tailored to the requirements of CpG clusters. In general, this distribution can be applied to experiments with dichotomous outcomes (either success or failure) and gives the probability of having a certain number of failures when the number of successes was fixed in advance, taking into account that the experiment must always end with a success. By translating these requirements to a genomic context, the successes were equated with CpG dinucleotides and the failures with non-CpGs (all other 15 possible dinucleotides). One prerequisite is that all trials be independent, which is not automatically fulfilled when dealing with overlapping dinucleotides (note that a CpG dinucleotide will always be followed by a non-CpG [GN] dinucleotide). Therefore, these "forced" non-CpGs need to be considered when calculating the success probabilities. Thus, the probability for a cluster with a number (*N*) of CpGs is given by

PN,p(nf)=(nf−(N+1)−1(N−1)−1)⋅pN−1⋅(1−p)nf     [3]
 MathType@MTEF@5@5@+=feaafiart1ev1aaatCvAUfKttLearuWrP9MDH5MBPbIqV92AaeXatLxBI9gBaebbnrfifHhDYfgasaacH8akY=wiFfYdH8Gipec8Eeeu0xXdbba9frFj0=OqFfea0dXdd9vqai=hGuQ8kuc9pgc9s8qqaq=dirpe0xb9q8qiLsFr0=vr0=vr0dc8meaabaqaciaacaGaaeqabaqabeGadaaakeaacqWGqbaudaWgaaWcbaGaemOta4KaeiilaWIaemiCaahabeaakiabcIcaOiabd6gaUnaaBaaaleaacqWGMbGzaeqaaOGaeiykaKIaeyypa0ZaaeWaaeaafaqabeGabaaabaGaemOBa42aaSbaaSqaaiabdAgaMbqabaGccqGHsislcqGGOaakcqWGobGtcqGHRaWkcqaIXaqmcqGGPaqkcqGHsislcqaIXaqmaeaacqGGOaakcqWGobGtcqGHsislcqaIXaqmcqGGPaqkcqGHsislcqaIXaqmaaaacaGLOaGaayzkaaGaeyyXICTaemiCaa3aaWbaaSqabeaacqWGobGtcqGHsislcqaIXaqmaaGccqGHflY1cqGGOaakcqaIXaqmcqGHsislcqWGWbaCcqGGPaqkdaahaaWcbeqaaiabd6gaUnaaBaaameaacqWGMbGzaeqaaaaakiaaxMaacaWLjaWaamWaaeaacqaIZaWmaiaawUfacaGLDbaaaaa@5F21@

This formula takes into account that all our clusters start with a CpG, and therefore the number of successes is *N-1 *(instead of *N*, the number of CpGs in the cluster). The number of independent non-CpGs (*n*_*f*_) in a cluster (failures) can be calculated as:

*n*_*f *_= *L *- 2 · *N *      [4]

*L *being the cluster length (in nucleotides). The success probability *p *(probability of finding a CpG) is calculated as:

p=Nsnis     [5]
 MathType@MTEF@5@5@+=feaafiart1ev1aaatCvAUfKttLearuWrP9MDH5MBPbIqV92AaeXatLxBI9gBaebbnrfifHhDYfgasaacH8akY=wiFfYdH8Gipec8Eeeu0xXdbba9frFj0=OqFfea0dXdd9vqai=hGuQ8kuc9pgc9s8qqaq=dirpe0xb9q8qiLsFr0=vr0=vr0dc8meaabaqaciaacaGaaeqabaqabeGadaaakeaacqWGWbaCcqGH9aqpdaWcaaqaaiabd6eaonaaBaaaleaacqWGZbWCaeqaaaGcbaGaemOBa42aaSbaaSqaaiabdMgaPjabdohaZbqabaaaaOGaaCzcaiaaxMaadaWadaqaaiabiwda1aGaay5waiaaw2faaaaa@3A88@

*N*_*s *_being the number of CpG dinucleotides in the sequence and *n*_*is *_the number of independent dinucleotides (i.e. including the CpGs but excluding the forced non-CpGs). The theoretical probabilities determined by this analytical method agree well with those found by numerical simulation, as shown in Additional file 6. Given its lower computational cost, the theoretical approach was implemented in our software.

The negative binomial is a two-tailed distribution (Additional file 6). The left tail indicates a high local CpG clustering (accumulation of CpGs) while the right tail is comprised of CpG-depleted regions. Therefore, the probability that an observed local CpG frequency is significantly higher than those expected under random conditions (CpG clustering) is given by the cumulative density function of the CpG cluster at point *n*_*f*_, which can therefore be taken as its *p-value*:

PN,pcum(x<=nf)=∑x=0nf(x−(N+1)−1(N−1)−1)⋅pN−1⋅(1−p)x     [6]
 MathType@MTEF@5@5@+=feaafiart1ev1aaatCvAUfKttLearuWrP9MDH5MBPbIqV92AaeXatLxBI9gBaebbnrfifHhDYfgasaacH8akY=wiFfYdH8Gipec8Eeeu0xXdbba9frFj0=OqFfea0dXdd9vqai=hGuQ8kuc9pgc9s8qqaq=dirpe0xb9q8qiLsFr0=vr0=vr0dc8meaabaqaciaacaGaaeqabaqabeGadaaakeaacqWGqbaudaqhaaWcbaGaemOta4KaeiilaWIaemiCaahabaGaem4yamMaemyDauNaemyBa0gaaOGaeiikaGIaemiEaGNaeyipaWJaeyypa0JaemOBa42aaSbaaSqaaiabdAgaMbqabaGccqGGPaqkcqGH9aqpdaaeWbqaamaabmaabaqbaeqabiqaaaqaaiabdIha4jabgkHiTiabcIcaOiabd6eaojabgUcaRiabigdaXiabcMcaPiabgkHiTiabigdaXaqaaiabcIcaOiabd6eaojabgkHiTiabigdaXiabcMcaPiabgkHiTiabigdaXaaaaiaawIcacaGLPaaacqGHflY1cqWGWbaCdaahaaWcbeqaaiabd6eaojabgkHiTiabigdaXaaakiabgwSixlabcIcaOiabigdaXiabgkHiTiabdchaWjabcMcaPmaaCaaaleqabaGaemiEaGhaaaqaaiabdIha4jabg2da9iabicdaWaqaaiabd6gaUnaaBaaameaacqWGMbGzaeqaaaqdcqGHris5aOGaaCzcaiaaxMaadaWadaqaaiabiAda2aGaay5waiaaw2faaaaa@6C76@

The use of this latter expression allows us to discriminate between the clusters found in the first step of the algorithm: those clusters with a *p-value *below a given threshold (usually 10^-5^, see Section "Benchmarking *CpGcluster*") are predicted as CGIs, while the rest of the clusters are discarded.

### Assembling test sequences containing CpG islands

To evaluate the accuracy of *CpGcluster *and compare it to other programs, we assembled a set of test sequences on the basis of an experimental CGI library [33]. This physical library was constructed using a two-step cloning strategy involving the isolation of GC-rich chromosomal fragments based on their lack of methylation *in vivo*, followed by an enrichment of fragments that could be methylated *in vitro*. The following steps were taken to assemble the test sequences:

(1) The full list of DerLab CGIs [33] was retrieved. These experimental CGIs can be quite short and the minimum length is actually 8. Out of the 6235 CGIs, 1612 (or 26 %) were shorter than 200 bp. The experimental islands were then divided into two groups: those that overlapped with the TSS and those that did not. The TSS coordinates were taken from the DBTSS database [34]. These two groups differed significantly in their mean length, CpG density, and CpG fraction, with all values being higher for the TSS group. In assembling the test sequences, we exclusively used the TSS group, which had a greater average length.

(2) In addition, non-island sequences – the sequences located between the CGIs of chromosome 22, as specified by the UCSC annotation [30] – were extracted.

(3) To further ensure a random background for the CGIs in our test sequences, all non-island segments were randomly shuffled using an algorithm that preserves dinucleotide frequencies [35]. As non-island segments could contain some non-annotated CGIs, this step ensures the randomness of the non-island segments, at the same time conserving nucleotide and dinucleotide compositions. This setup is the less biased, as none of the finders is expected to predict CGIs on randomized sequences.

(4) The shuffled non-island segments were then alternatively combined with 400 island segments overlapping with TSS, chosen at random from the DerLab sample, thus assembling a test sequence of approximately 18 Mb in length.

(5) Using the assembling process described in step (4), we generated a set of 10 test sequences containing experimental CGIs alternating with shuffled non-island segments.

### Availability and requirements

Project name: CpGcluster

Project home pages: 



Operating system(s): platform independent

Programming language: Perl 5 (see Additional file 7 for source code)

Licence: open source

## Abbreviations

CC: Correlation Coefficient

CGI: CpG island

CpG O/E ratio: Ratio between observed and expected CpG frequencies

CpG: dinucleotide CG

G+C content, %G+C: Molecular fraction of guanine and cytosine

NG: non-genic islands

PhastCons: Phylogenetic Conserved Elements

Sn: Sensitivity – the proportion of island nucleotides which have been correctly predicted as islands

Sp: Specificity – the proportion of predicted island nucleotides that are actually islands

SPR: promoter of SERPINB5 gene

TES: Transcription End Site

TSS: Transcription Start Site

UTR: Untranslated Regions

## Authors' contributions

JLO proposed the distance approach, then associating a *p-value *with each predicted island by means of a randomization test. JMA and PLE wrote and analyzed a prototype program based on distances and suggested to assign *p-values *analytically. MH proposed the use of the negative binomial distribution for computing *p-values*, carried out the randomization tests and implemented the final version into a Perl script. CP evaluated the accuracy of *CpGcluster *against other island-finding programs. PC carried out the analysis of the CpG distance geometric distribution which motivates the use of the median distance as a threshold. JLO, MH and PC drafted the manuscript and edited the contributions from the remaining authors. All the authors read and approved the final version.

## Supplementary Material

Additional file 1 Alignment with SERPINB5 promoter. Sequence alignment between the promoter of SERPINB5 (SPR, associated with a GC-rich region) and the CGI predicted by CpGcluster. None of the remaining CGI finders were able to detect any CGI in this region. The transcription start site (TSS), as given by the DBTSS data base [34], is shown in bold type. Click here for file

Additional file 2 Distribution of distances between neighboring CpG dinucleotides in the human chromosomes 1 to 6 Click here for file

Additional file 3 Distribution of distances between neighboring CpG dinucleotides in the human chromosomes 7 to 12 Click here for file

Additional file 4 Distribution of distances between neighboring CpG dinucleotides in the human chromosomes 13 to 18 Click here for file

Additional file 5 Distribution of distances between neighboring CpG dinucleotides in the human chromosomes 19 to Y Click here for file

Additional file 6 Comparison between analytical and experimental randomization. Length distribution of clusters with different numbers of CpGs. The analytical distribution (black line) is virtually identical to the experimental one obtained by a randomization that preserves the CpG frequency. Click here for file

Additional file 7 Program source code (Perl) Click here for file
