# Supplementary material for: CpGcluster: a distance-based algorithm for CpG-island detection
Source: BMC Bioinformatics. 2006 Oct 12;7:446. doi: 10.1186/1471-2105-7-446 (PMC1617122; doi:10.1186/1471-2105-7-446)
Supplement: Additional file 1 — Alignment with SERPINB5 promoter. Sequence alignment between the promoter of SERPINB5 (SPR, associated with a GC-rich region) and the CGI predicted by CpGcluster. None of the remaining CGI finders were able to detect any CGI in this region. The transcription start site (TSS), as given by the DBTSS data base [34], is shown in bold type. [file 1471-2105-7-446-S1.doc]

CGI -------------------------------------------------- 0

SPR TGCAGTTTACACAAAAAGAATGGAGATCAGAGTACTTTTTGTGCCACCAA 50

CGI -------------------------------------------------- 0

SPR CGTGTCTGAGAAATTTGTAGTGTTACTATCATCACACATTACTTTTATTT 100

CGI -------------------------------------------------- 0

SPR CATCGAATATTTCACCTTCCGGTCCTGCGTGGGCCGAGAGGATTGCCGTA 150

CGI -------------------------------------------------- 0

SPR CGCATGTCTGTACGTATGCATGTAACTCACAGCCCCTTCCTGCCCGAACA 200

CGI -------------------------------------------------- 0

SPR TGTTGGAGGCCTTTTGGAAGCTGTGCAGACAACAGTAACTTCAGCCTGAA 250

CGI -------------------------------------------------- 0

SPR TCATTTCTTTCAATTGTGGACAAGCTGCCAAGAGGCTTGAGTAGGAGAGG 300

CGI -----CGCCGAGGCGGGGCGGGGCGGGGCGTGGAGCTGGGCTGGCAGTGG 45

SPR AGTGCCGCCGAGGCGGGGCGGGGCGGGGCGTGGAGCTGGGCTGGCAGTGG 350

*********************************************

CGI GCGTGGCGGTGCTGCCCAGGTGAGCCACCGCTGCTTCTGCCCAGACACGG 95

SPR GCGTGGCGGTGCTGCCCAGGTGAGCCACCGCTGCTTCTGCCCAGACACGG 400

**************************************************

CGI TCGCCTCC**A**CATCCAGGTCTTTGTGCTCCTCGCTTGCCTGTTCCTTTTCC 145

SPR TCGCCTCC**A**CATCCAGGTCTTTGTGCTCCTCGCTTGCCTGTTCCTTTTCC 450

******************************************************

CGI ACG---------------------------- 148

SPR ACGCATTTTCCAGGATAACTGTGACTCCAGG 481

***
