# Supplementary material for: CpGcluster: a distance-based algorithm for CpG-island detection
Source: BMC Bioinformatics. 2006 Oct 12;7:446. doi: 10.1186/1471-2105-7-446 (PMC1617122; doi:10.1186/1471-2105-7-446)
Supplement: Supplementary file 2 [file 1471-2105-7-446-S2.doc]

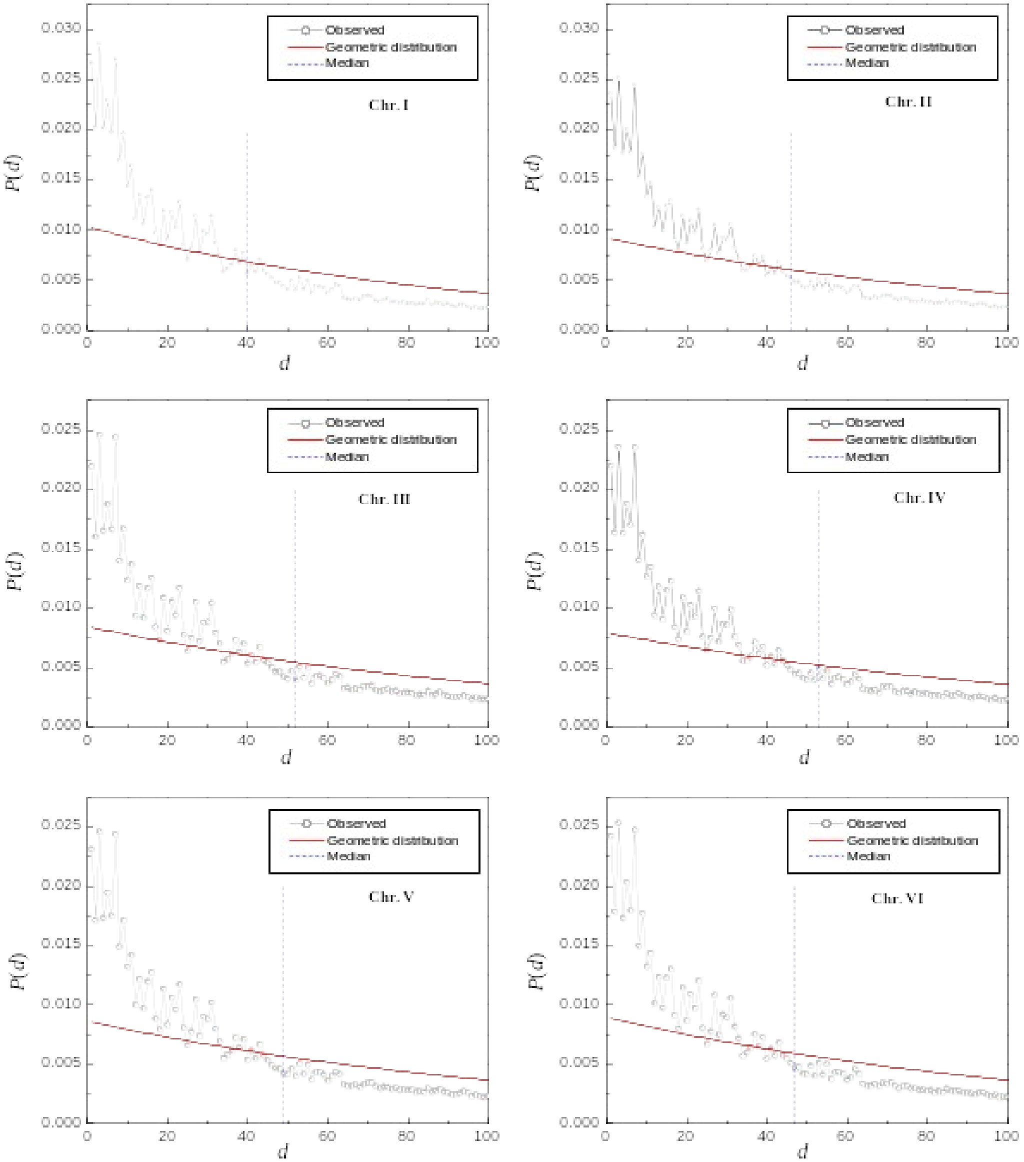
Observed
Geometric distribution
Median
Chr. I
Chr. II
Chr. III
Chr. IV
Chr. V
Chr. VI
P(d)
d
0.000
0.005
0.010
0.015
0.020
0.025
0.030
0
20
40
60
80
100
