# Supplementary material for: CpGcluster: a distance-based algorithm for CpG-island detection
Source: BMC Bioinformatics. 2006 Oct 12;7:446. doi: 10.1186/1471-2105-7-446 (PMC1617122; doi:10.1186/1471-2105-7-446)
Supplement: Supplementary file 4 [file 1471-2105-7-446-S4.doc]

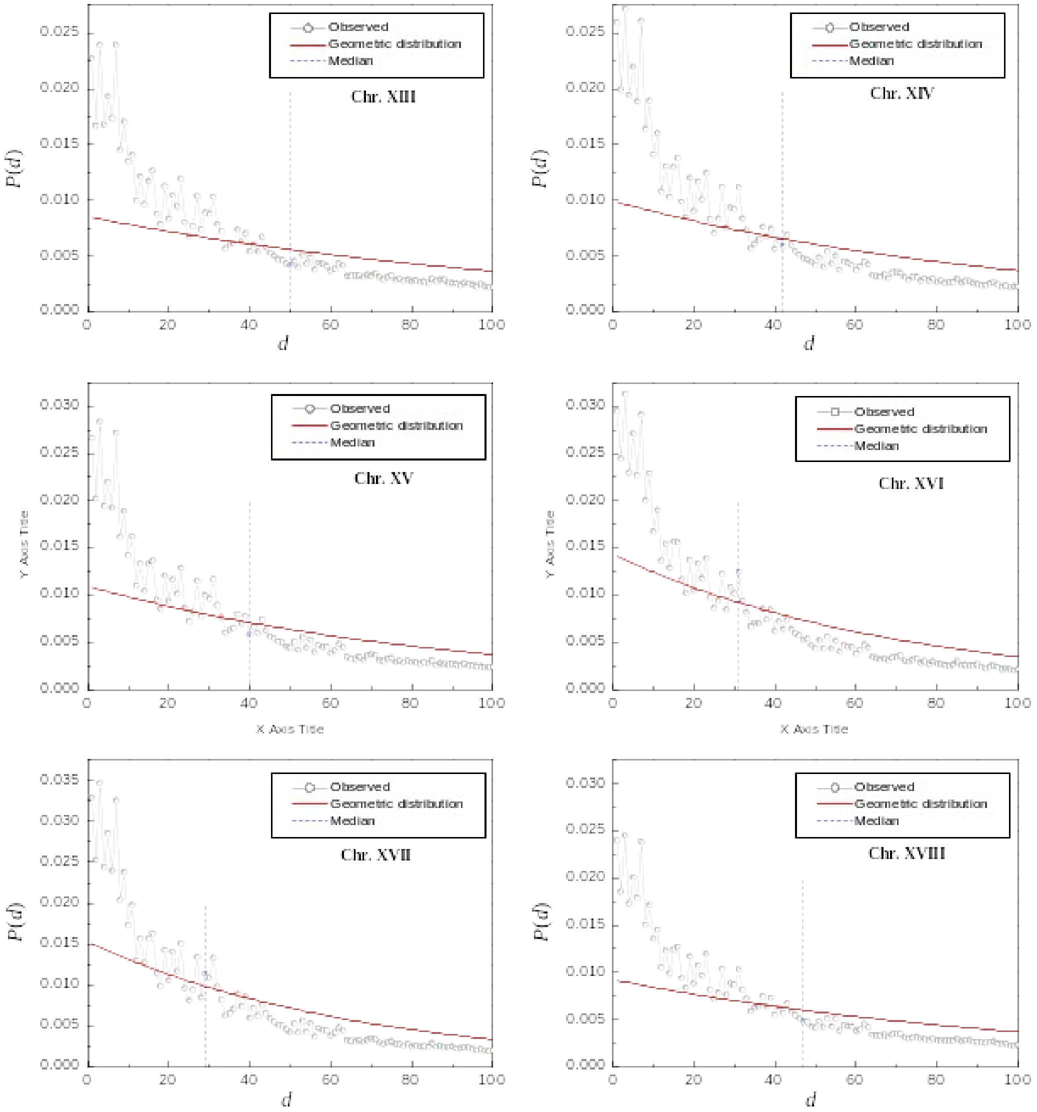

Observed
Geometric distribution
Median
Chr. XIII
Chr. XIV
Chr. XV
Chr. XVI
Chr. XVII
Chr. XVIII
$P(d)$
d
Y Axis Title
X Axis Title
