# Supplementary material for: CpGcluster: a distance-based algorithm for CpG-island detection
Source: BMC Bioinformatics. 2006 Oct 12;7:446. doi: 10.1186/1471-2105-7-446 (PMC1617122; doi:10.1186/1471-2105-7-446)
Supplement: Supplementary file 5 [file 1471-2105-7-446-S5.doc]

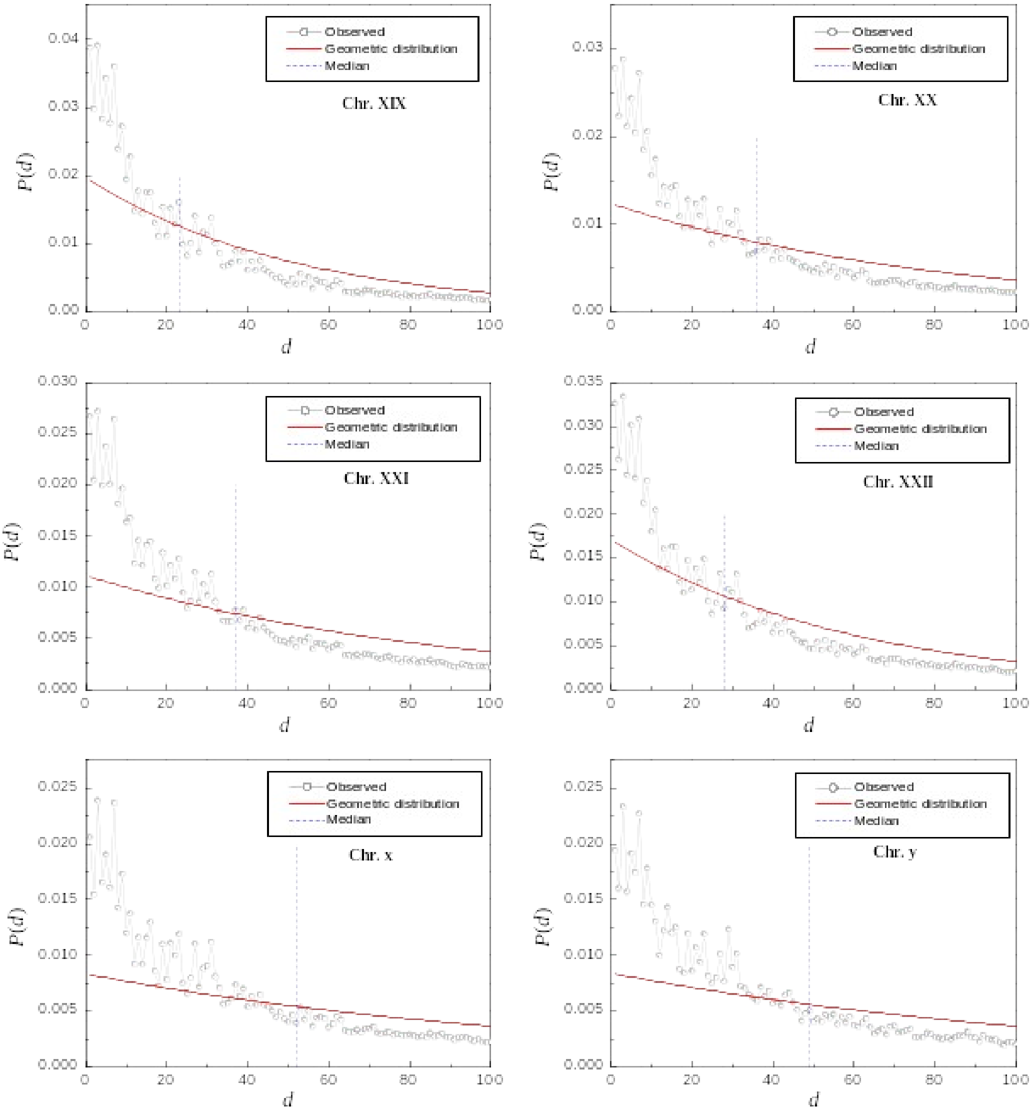

Observed
Geometric distribution
Median
Chr. XIX
Chr. XX
Chr. XXI
Chr. XXII
Chr. x
Chr. y
P(d)
d
