# Supplementary material for: CpGcluster: a distance-based algorithm for CpG-island detection
Source: BMC Bioinformatics. 2006 Oct 12;7:446. doi: 10.1186/1471-2105-7-446 (PMC1617122; doi:10.1186/1471-2105-7-446)
Supplement: Supplementary file 6 [file 1471-2105-7-446-S6.doc]

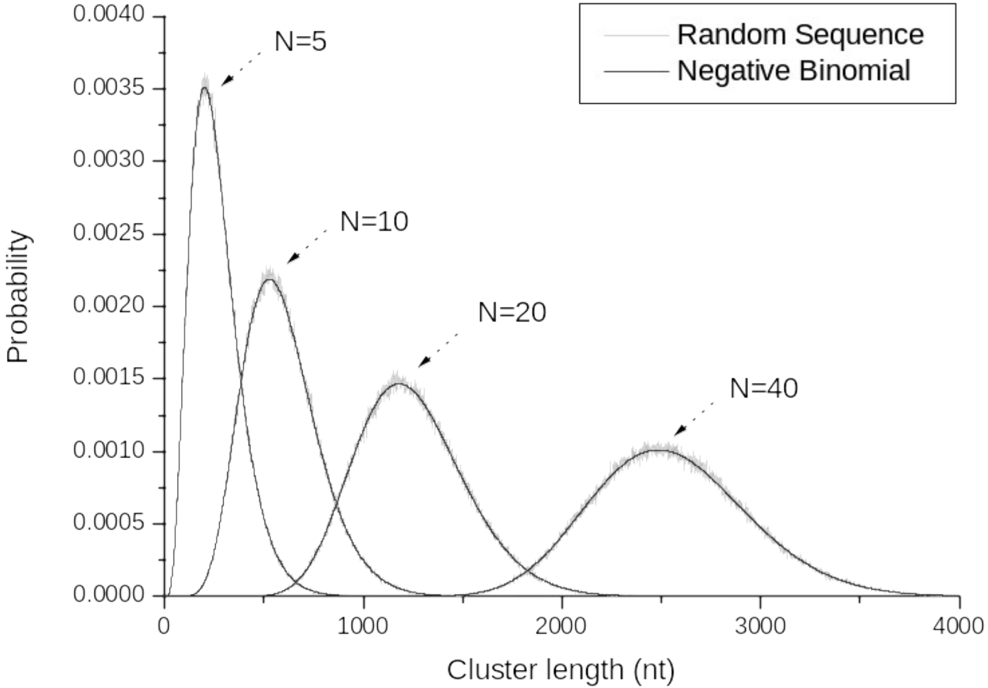

Random Sequence
Negative Binomial
N=5
N=10
N=20
N=40
Probability
0.0040
0.0035
0.0030
0.0025
0.0020
0.0015
0.0010
0.0005
0.0000
0
1000
2000
3000
4000
Cluster length (nt)
